# Controlling Chronic Diseases Through Evidence-Based Decision Making: A Group-Randomized Trial

**DOI:** 10.5888/pcd14.170326

**Published:** 2017-11-30

**Authors:** Ross C. Brownson, Peg Allen, Rebekah R. Jacob, Anna deRuyter, Meenakshi Lakshman, Rodrigo S. Reis, Yan Yan

**Affiliations:** 1 Prevention Research Center in St. Louis, Brown School, Washington University in St. Louis, St. Louis, Missouri; 2 Department of Surgery, Division of Public Health Sciences, and Alvin J. Siteman Cancer Center, Washington University School of Medicine, Washington University in St. Louis, St. Louis, Missouri; 3 Division of Biostatistics, Washington University School of Medicine, Washington University in St. Louis, St. Louis, Missouri

## Abstract

**Introduction:**

Although practitioners in state health departments are ideally positioned to implement evidence-based interventions, few studies have examined how to build their capacity to do so. The objective of this study was to explore how to increase the use of evidence-based decision-making processes at both the individual and organization levels.

**Methods:**

We conducted a 2-arm, group-randomized trial with baseline data collection and follow-up at 18 to 24 months. Twelve state health departments were paired and randomly assigned to intervention or control condition. In the 6 intervention states, a multiday training on evidence-based decision making was conducted from March 2014 through March 2015 along with a set of supplemental capacity-building activities. Individual-level outcomes were evidence-based decision making skills of public health practitioners; organization-level outcomes were access to research evidence and participatory decision making. Mixed analysis of covariance models was used to evaluate the intervention effect by accounting for the cluster randomized trial design. Analysis was performed from March through May 2017.

**Results:**

Participation 18 to 24 months after initial training was 73.5%. In mixed models adjusted for participant and state characteristics, the intervention group improved significantly in the overall skill gap (*P* = .01) and in 6 skill areas. Among the 4 organizational variables, only access to evidence and skilled staff showed an intervention effect (*P* = .04).

**Conclusion:**

Tailored and active strategies are needed to build capacity at the individual and organization levels for evidence-based decision making. Our study suggests several dissemination interventions for consideration by leaders seeking to improve public health practice.

## Introduction

An evidence-based approach to chronic disease prevention and control can significantly reduce the burden of chronic diseases (1). Large-scale efforts such as Cancer Control P.L.A.N.E.T. (https://cancercontrolplanet.cancer.gov/) and the *Community Guide* placed various evidence-based interventions in the hands of cancer control practitioners (2). Even with knowledge of effective interventions, often 15 to 20 years elapse before research findings are incorporated into practice (3). Knowledge of effective approaches for dissemination of evidence-based interventions is growing (4,5). Practitioners in state health departments can assess a public health problem, develop an appropriate program or policy to address the problem, and ensure that programs and policies are effectively delivered and implemented (6).

The process of evidence-based decision making (EBDM) involves multiple elements, including making decisions that are based on the best available scientific or rigorous evaluation evidence, applying program planning and quality improvement frameworks, engaging the community in assessment and decision making, adapting and implementing evidence-based interventions for specific populations or settings, and conducting sound evaluation (7). To select and implement evidence-based interventions in diverse populations and settings, advanced knowledge and skill is needed in key processes (eg, adaptation of interventions, evaluation) (8).

Previous research with state health agencies showed that although levels of awareness of EBDM is high, implementation of evidence-based interventions varies widely and is limited in many states (9). Similarly, another study found that although cancer control practitioners showed a strong preference for programs with proven effectiveness, fewer than half of respondents in that study (48%) had ever used resources on evidence-based interventions (10). A national survey of state practitioners in chronic disease control found that only 20% used evidence-based interventions often in their work (11). Nonetheless, staff members in state public health agencies recognize the need for capacity building to support implementation of effective practices (10).

Putting evidence to use in public health settings requires sufficient capacity — the availability of resources, structures, and workforce to deliver the preventive dose of an evidence-based intervention (12). Capacity is a determinant of performance; that is, greater capacity is linked with greater effect on public health (13,14). Success in implementing EBDM in public health settings is achieved by building the skills of individuals (eg, their ability to carry out a program evaluation) and organizations (eg, achieving a climate and culture that supports innovation and evidence-based approaches) (12). These 2 skills are interrelated in that individuals shape organizations and organizations support the development of individuals (15).

To date, little research has addressed the most effective approaches for building capacity for EBDM in state public health agencies seeking to address chronic disease prevention and control. The objective of this study was to test whether providing training and other support to state health departments increased the use of EBDM processes to prevent chronic diseases at both the individual level (eg, reducing skill gaps) and the organization level (eg, increasing participatory decision making).

## Methods

We conducted a 2-arm, group-randomized trial consisting of an intervention arm and a control arm (Figure). We assessed 50 states and the District of Columbia for eligibility. We excluded 3 states with the lowest burden of cancer and overall chronic disease, 3 states with the lowest capacity for EBDM, 2 states with the highest capacity for EBDM, 7 states that had already received extensive EBDM training, and 3 states that had no logical pair match. State exclusion criteria are detailed elsewhere (16). The remaining 33 states were organized into tertiles according to state population size. Two pairs from each state population tertile were selected in 3 rounds of staggered selection and enrollment. Each round consisted of 1 state randomly selected from each of 2 tertiles and matched with the nearest population-sized state within the tertile. Six state health department’s chronic disease prevention units (hereinafter called states) were selected via a simple randomization method by our data analyst (R.R.J.) and then pair-matched with the state closest in population size, to decrease between-state variability, for a total of 6 pairs (6 intervention states and 6 control states, 1 each per pair). We then invited the states to participate by contacting the chronic disease director in each state health department. Two states declined to participate, and we selected the state with the nearest population in the tertile to replace that state to retain our total of 12. After pairing and obtaining consent from the lead chronic disease official, whom we designated as the state-level representative, the 2 states in each pair were randomly assigned to the intervention arm or control arm. There was no blinding. Enrollment of state pairs, data collection, and intervention trainings were staggered for scheduling feasibility. Enrollment of states took place from September 2013 through May 2014. The trial was registered with ClinicalTrials.gov (NCT01978054) (17). The study was approved by the Washington University in St. Louis Institutional Review Board (no. 201111105).

**Figure:**
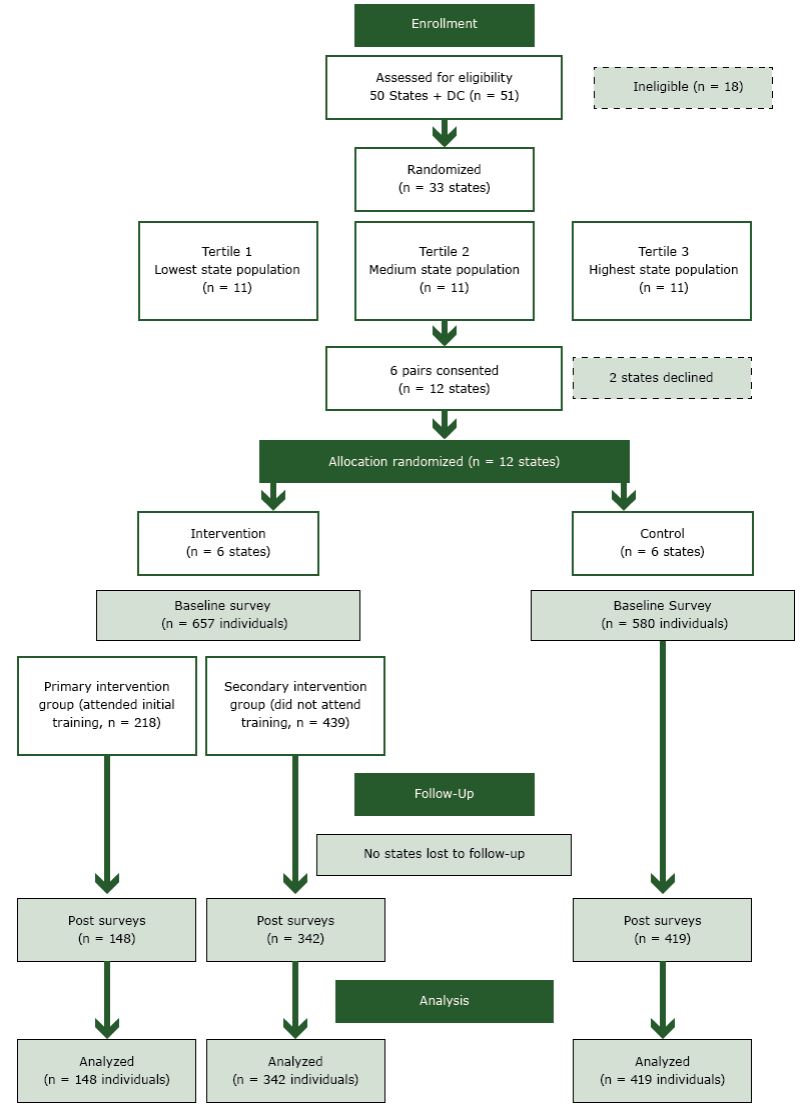
Flow diagram of the study of evidence-based decision making conducted in 12 states, 2014–2016 (CONSORT diagram).

### Intervention

**Intervention states.** The intervention began with a 3.5-day training in EBDM conducted onsite at each of the 6 intervention states between March 2014 and March 2015. Training details are described elsewhere (18). The lead official responsible for chronic disease control in each state assisted the team in recruiting training participants from among their staffs and sometimes included staff members from state or local partnering organizations. A total of 222 staff members attended a multiday EBDM course in 1 of the 6 intervention states. All intervention state participants were asked to complete an online baseline survey before the multiday training. Each intervention state received a report on its baseline survey results for planning purposes and selected supplemental capacity-building activities, typically brief trainings or management strategies intended to build an organizational culture of EBDM, improve staff access to research evidence, share information, and build evaluation capacity (Appendix Table 1). Follow-up conference calls with intervention states provided technical assistance and supplemental activity planning and updates.

**Control states** *.* Control states identified participants for data collection and received a list of EBDM resources, web links, and state-specific baseline and post-intervention findings. They received no training, and all control state participants were asked to complete an online baseline survey before their paired state’s multiday training.

### Participants

Study participants were 2 groups of chronic disease control practitioners at the state and local level, an intervention group and a control group. These were people who directed and implemented population-based intervention programs in government agencies or in community-based coalitions. Participants were directly involved in delivering programs, setting priorities, or allocating resources for programs related to chronic disease risk factors or screening. Examples were the director of a comprehensive chronic disease program for the state or a leader in a state or regional chronic disease control coalition. 

The intervention arm comprised 2 groups: a primary group and a secondary group. The primary group in each intervention state was made up of staff members who attended the EBDM course; most worked in state health departments and a few were from state or local partnering organizations. The secondary group in each intervention state, none of whom attended the EBDM course, was made up of chronic disease staff members and partnering staff members from each state health department, local health departments, universities, and coalitions (collaborators). Collaborators were surveyed because they were expected to apply EBDM in their organizations for control of chronic diseases as funded or guided by the state. Inclusion of collaborators also helped the study team meet sample size requirements. All participants were aged 20 years or older and able to take an online survey in English. Across the entire sample, most participants worked either in chronic disease risk reduction or chronic disease screening.

### Measures, data collection, and statistical analysis

Measures in the 65-item online Qualtrics Version January 2014–November 2016 (Qualtrics) survey were informed by a literature review (13) and earlier research by the study team (16,19). Measures, described in detail elsewhere (16,20,21), were tested with cognitive response methods and test–retest reliability (16). Survey questions assessed individual-level skills (eg, adapting interventions, action planning, communicating to policy audiences) and organizational-level capacities (eg, access to evidence, program evaluation, perceived supervisory expectations) (Appendix Table 2). Survey participants were asked to rate on a 11-point Likert scale the perceived importance and perceived availability of 10 EBDM skills.

Online self-report surveys were administered, by state, at 2 points at staggered times: 1) a baseline survey conducted from January 2014 through December 2014 and 2) a post-intervention survey conducted from October 2015 through November 2016, 18 to 24 months after the state pair’s EBDM training. The study team followed up each returned post-survey email invitation to determine whether the participant had left the agency or just had a new email address and recorded reasons for declining among those who declined the post-survey by telephone or email. 

The unit of analysis was individual staff members, with individuals from all 12 clusters (states) who completed both surveys included in analyses. We calculated baseline intra-cluster correlations for the dependent variables using standard methods to assess need for mixed modeling, but we elected to conduct mixed modeling as a conservative approach regardless of result. One-stage mixed analysis of covariance (ANCOVA) models were fitted by using PROC MIXED (SAS Inc) with state as a random effect to account for clustering by state (22). The between–within method was used to calculate denominator degrees of freedom for the fixed effect instead of the SAS default containment method, because it is more appropriate for unbalanced study designs. SAS version 9.4 was used for descriptive analyses and mixed ANCOVA modeling, and SPSS version 24 (IBM Corp) was used to clean and recode data and create calculated variables. Covariates were included in final ANCOVA models when the unadjusted effect size was attenuated by 10% or more on the basis of addition of a particular covariate to the model (23). Sex was included in all adjusted models as required in studies funded by the National Institutes of Health. All tests of significance were 2-sided, including the χ^2^ tests and independent samples *t* tests used to compare baseline participant characteristics and scores. The sample size calculation of the study is described elsewhere (16).

The primary individual-level outcomes were gaps in EBDM skills among public health practitioners and their use of research evidence for job tasks. The primary organization-level outcomes were access to research evidence and the presence of a staff with EBDM skills, supervisory expectations for EBDM use, evaluation, and work unit participatory decision making as assessed through individuals’ perceptions. The main analyses compared data on the primary intervention arm participants with data on control participants; we also compared data on secondary intervention arm participants and control participants. 

We calculated gaps in the 10 EBDM skill scores by subtracting the score in perceived availability from the score in perceived importance for each individual for each skill. Higher gap scores indicate larger gaps. A summary score for gaps in skills was calculated for each individual by summing the values for gaps in scores for the 10 EBDM skills. A summary frequency of use of research evidence for job tasks was the calculated mean of the 6 job task responses.

We used items from a 7-point Likert scale (1 = “strongly disagree” and 7 = “strongly agree”) to conduct exploratory factor analysis with orthogonal rotation to create individual scores for 5 factors: 1) access to research evidence and resources (4 items), 2) evaluation capacity (3 items); 3) supervisory expectations (3 items), 4) participatory decision making (3 items), and 5) agency leadership support (3 items) as in a previous national survey with state health department public health practitioners (21). By definition, the factor scores had a mean of zero and were normally distributed. One or more organization behavior items were left blank by 34 of the 567 survey participants (6.0%); these participants were excluded from factor score creation and mixed ANCOVA modeling.

## Results

At baseline, 1,237 of the 1,508 invited public health practitioners completed the online survey (82.0% response, 83.6% for the 6 intervention states, 80.2% for controls). At follow-up, 909 (73.5%) of baseline participants completed the post-intervention survey, with a median of 73 participants per state (mean, 75.8; standard deviation [SD], 10.6). Loss to survey follow-up was primarily due to staff turnover. Of the 222 people assigned to the primary intervention arm who attended the EBDM training, 148 (66.7%) completed both baseline and post-intervention surveys (Table 1); of the 439 secondary intervention arm participants, 342 (77.9%) completed both surveys, and of the 580 control participants, 419 (72.2%) completed both surveys. Overall, most baseline survey participants were women (80.6%), and 64.3% had at least a master’s degree in any field. At baseline, primary intervention participants differed significantly from control participants in several characteristics: for example, the percentage working in state health departments, age, and the percentage holding a master’s degree or doctorate in public health. The number of primary intervention arm participants varied by state from 18 to 32, and the number of control participants varied by state from 65 to 72. 

**Table 1:** Characteristics of Participants at Baseline Among Primary Intervention Participants and Controls in 12 States, Study of Evidence-Based Decision Making, 2014–2015^a^

Characteristic	Overall (n = 567)	Primary Intervention Group^b^ (n = 148)	Control Group^c^ (n = 419)	*P *Value^d^
**State health department**	56.6	81.8	47.7	<.001
**Position type**				
Leadership position	17.0	16.9	17.0	.74
Program manager or coordinator	48.2	50.0	47.6	
Health specialist	30.6	30.4	30.6	
Other type specified	4.2	2.7	4.8	
Female	80.6	84.4	79.3	.18
**Age, y**				
20–29	5.4	10.3	3.6	.02
30–39	20.4	23.3	19.4	
40–49	26.1	24.0	26.9	
50–59	33.1	30.1	34.1	
≥60	15.0	12.3	16.0	
**Education**				
Master’s degree or higher in any field	64.3	64.9	64.1	.86
Public health master’s degree or doctorate	22.5	35.8	17.7	<.001
Nursing degree	10.5	11.9	10.2	.66
**Chronic disease prevention and control revenue from CDC as of October 2014, in millions of dollars, mean (SD)**	14.5 (7.6)	16.8 (9.4)	13.7 (6.7)	<.001
**Size of state population, by tertile**				
Small	32.1	34.5	31.3	.28
Mid-size	34.9	37.8	33.9	
Large	33.0	27.7	34.8	
**Percentage of state population living in urban area, mean (SD)**	68.9 (15.2)	67.4 (10.1)	69.4 (16.6)	.09
**Percentage of state population living in poverty, mean (SD)**	14.6 (4.2)	12.4 (3.8)	15.4 (4.0)	<.001
**State political party control in 2014 of governorship, state house, state senate**				
All Republican control	51.8	51.4	52.0	<.001
Divided party control	27.3	12.8	32.5	
All Democratic control	20.8	35.8	15.5	

Abbreviation: CDC, Centers for Disease Control and Prevention; SD, standard deviation.

a Values are percentages unless otherwise indicated. Only participants who completed the baseline survey and follow-up survey (18 to 24 months later) were included in the analysis.

b Intervention arm comprised a primary group, which attended an evidenced-based decision-making course, and a secondary group, which did not attend an evidenced-based decision-making course but participated in other training activities.

c Control group received no training.

d *P* values calculated by using 2-sided χ^2^ or *t* test to test differences between primary intervention group and control group.

The largest EBDM skill gaps at baseline were for adapting interventions, economic evaluation, and communicating research to policy makers (Table 2). Mean scores at baseline did not differ significantly between groups, except for 3 skills: adapting interventions (*t* = 2.49, *P* = .01), economic evaluation (*t* = 2.10, *P* = .04), and community assessment (*t* = 2.01, *P* = .04). Baseline intra-cluster correlations were low in all the states (ranging from <.001 to .018), indicating low correlation of responses within states.

**Table 2:** Mean Scores at Baseline and Post-Intervention in 12 States, Study of Evidence-Based Decision Making, 2014–2016

Dependent Variable	Primary Intervention Group^a^, Mean (95% CI) (n = 148)		Control Group^b^, Mean (95% CI) (n = 419)		*P *Value^c^
	Baseline	Post-Intervention	Baseline	Post-Intervention	
**Individual Capacity**					
**EBDM skill gap^d^ (10-item sum)**	20.4 (17.8 to 23.1)	15.3 (12.8 to 17.9)	18.3 (16.6 to 19.9)	17.6 (16.0 to 19.1)	.17
Prioritization	1.7 (1.4 to 2.0)	1.0 (0.7 to 1.3)	1.7 (1.5 to 1.8)	1.4 (1.2 to 1.6)	.79
Adapting interventions	2.6 (2.2 to 2.9)	1.8 (1.5 to 2.2)	2.0 (1.8 to 2.2)	2.1 (1.8 to 2.3)	.01
Quantifying the issue	1.5 (1.1 to 1.9)	0.9 (0.6 to 1.3)	1.4 (1.2 to 1.6)	1.4 (1.2 to 1.6)	.71
Evaluation designs	2.1 (1.7 to 2.6)	1.6 (1.2 to 2.0)	1.9 (1.7 to 2.1)	1.9 (1.7 to 2.1)	.34
Quantitative evaluation	1.3 (0.9 to 1.6)	1.0 (0.7 to 1.3)	1.3 (1.1 to 1.5)	1.3 (1.1 to 1.5)	.99
Qualitative evaluation	1.9 (1.5 to 2.3)	1.4 (1.0 to 1.8)	1.7 (1.5 to 1.9)	1.7 (1.5 to 2.0)	.32
Economic evaluation	3.5 (3.0 to 4.0)	3.5 (3.0 to 4.0)	2.9 (2.6 to 3.2)	2.8 (2.528 to 3.1)	.04
Action planning	1.3 (1.0 to 1.6)	0.9 (0.6 to 1.2)	1.2 (1.1 to 1.4)	1.0 (0.8 to 1.2)	.88
Community assessment	1.9 (1.6 to 2.2)	1.3 (1.0 to 1.7)	1.5 (1.3 to 1.7)	1.5 (1.3 to 1.7)	.04
Communicating research to policy makers	2.5 (2.1 to 3.0)	1.9 (1.5 to 2.3)	2.6 (2.3 to 2.9)	2.5 (2.2 to 2.8)	.73
**Use of research evidence for job tasks (6-item mean)^e^**	1.8 (1.7 to 2.0)	2.0(1.9 to 2.1)	1.9 (1.8 to 2.0)	1.9 (1.9 to 2.0)	.52
**Organizational Capacity^f^**					
Access to evidence and skilled staff (4-item factor)	−0.1 (−0.2 to 0.1)	0.2 (0.0 to 0.3)	0.1 (−0.0 to 0.2)	−0.1 (−0.2 to 0.0)	.08
Program evaluation (3-item factor)	−0.0 (−0.2 to 0.1)	0.0 (−0.1 to 0.2)	0.1 (−0.0 to 0.2)	0.1 (−0.0 to 0.1)	.30
Supervisory expectations and incentives factor (3-item factor)	0.1 (−0.0 to 0.3)	0.2 (0.1 to 0.3)	0.0 (−0.1 to 0.1)	0.1 (−0.0 to 0.2)	.34
Participatory decision making factor (3-item factor)	0.1 (−0.1 to 0.2)	−0.1 (−0.2 to 0.1)	−0.0 (−0.1 to 0.1)	0.0 (−0.1 to 0.1)	.28

a Intervention arm comprised a primary group, which attended an evidenced-based decision-making course, and a secondary group, which did not attend an evidenced-based decision-making course but participated in other training activities.

b Control group received no training.

c *P* values at baseline calculated by using independent samples *t* test (2 sided). Test compares gaps at baseline between primary intervention group and control group.

d Survey participants were asked to rate on a 11-point Likert scale the perceived importance and perceived availability of 10 EBDM skills; higher scores indicate larger gaps. We calculated gaps in the 10 EBDM skill scores by subtracting the score in perceived availability from the score in perceived importance for each individual for each skill. Observed skill gap scores ranged from −9 to +10 for specific skills and from −66 to +88 for the 10-item summed skill gap.

e Frequency of research evidence use scores ranged from 0 to 3 for each of 6 job tasks: 3 = weekly, 2 = monthly, 1 = quarterly, and 0 = seldom or never. A mean score for the 6 job tasks was calculated for each individual and could range from 0 to 3. The group mean frequencies shown in the table are lower than 2 = monthly.

f Organizational capacity variables shown here are the group means of the individual factor scores derived from exploratory factor analysis. For the sample overall, each factor by definition has a mean score of 0 and can range from -3 to +3. Observed group means are close to zero, either slightly below or above the overall sample mean of zero.

The primary intervention group significantly improved in the overall skill gap (*P* = .01) and in 6 skill areas compared with the control group (Table 3). In the comparison of secondary intervention arm participants and control participants, intervention effects on the 10 skill gaps were attenuated and no longer significant. 

**Table 3:** Intervention Effects at the Individual and Organization Levels Adjusted^a^ for Participant and State Characteristics in 12 States, Study of Evidence-Based Decision Making (EBDM),2014–2016

Dependent Variable	Intervention Effect Parameter Estimate^b^			
	β (SE)	95% Confidence Interval	*t*	*P *Value^b^
**Individual**				
**EBDM skill gaps (10-item sum)**	−5.56 (1.59)	−9.32 to −1.80	−3.50	.01
Prioritization	−0.58 (0.20)	−1.07 to −0.09	−2.89	.03
Adapting interventions	−0.69 (0.22)	−1.21 to −0.17	−3.13	.02
Quantifying the issue	−0.59 (0.22)	−1.09 to −0.08	−2.69	.03
Evaluation designs	−0.43 (0.24)	−1.00 to 0.14	−1.79	.11
Quantitative evaluation	−0.23 (0.21)	−0.77 to 0.26	−1.21	.33
Qualitative evaluation	−0.59 (0.24)	−1.19 to 0.02	−2.48	.05
Economic evaluation	0.18 (0.28)	−0.51 to 0.87	0.65	.54
Action planning	−0.35 (0.24)	0.91 to 0.20	−1.50	.18
Community assessment	−0.59 (0.22)	−1.11 to −0.06	−2.65	.03
Communicating research to policy makers	−0.96 (0.28)	−1.63 to −0.29	−3.41	.01
**Use of research evidence for job tasks (6-item mean)**	0.12 (0.07)	−0.04 to 0.28	1.74	.12

**Organization**				
Access to evidence and skilled staff (4- item factor)	0.37 (0.14)	0.02 to 0.72	2.73	.04
Program evaluation factor (3-item factor)	0.03 (0.10)	−0.21 to 0.26	0.28	.78
Supervisory expectations for EBDM (3-item factor)	−0.06 (0.26)	−0.73 to 0.62	−0.21	.84
Participatory decision making (3-item factor)	−0.06 (0.12)	−0.36 to 0.23	−0.57	.59

Abbreviation: SE, standard error.

a Participant characteristics were sex, agency, job position, age group, having a public health master’s or doctoral degree, and having a master’s or doctoral degree in any field; state characteristics were accreditation status, chronic disease revenue from the Centers for Disease Control and Prevention to the state public health department, tertile of state population size, percentage of state population living in urban area, percentage of state population living in poverty, and state party control of the governorship, state senate, and state house.

b Mixed analysis of covariance (ANCOVA) models with state as a random effect; parameter estimate *P* values are fixed solution 2-sided *t* tests within mixed ANCOVA.

Sex was included in all adjusted mixed ANCOVA models and did not affect sizes of intervention effects. Sex was not associated with gaps in skills, except that men were more likely than women to have a smaller gap in qualitative evaluation when we adjusted for other characteristics (β = −0.55, *t* = −2.45, *P* = .03). Having at least a master’s degree in any field was associated with increased use of research evidence for job tasks (β = 0.18, *t* = 2.93, *P* = .01) and with increased supervisory expectations of EBDM use (β = 0.24, *t* = 2.46, *P* = .03). Being in a leadership position was associated with increased participatory decision making, compared with the reference group of program managers (β = 0.27, *t* = 2.22, *P* = .03). Other participant and state characteristics did not affect the models. Among the 4 organizational capacity dependent variables, only access to evidence and skilled staff showed an intervention effect (*t* = 2.73, *P* = .04). In the comparison of secondary intervention arm participants and control participants, no intervention effects were significant for organization-level outcomes.

## Discussion

Our study is among the first to test the effects of strategies to increase the use of EBDM processes among public health practitioners engaged in controlling chronic diseases. We sought to reduce the gap between the generation of evidence and its application in practice settings, which can be viewed as “leakage in the dissemination pipeline” from discovery to application (24). In large part, this leakage relates to lack of individual and organizational capacity to practice EBDM (12).

Our 12-state study showed improvements in individual-level capacity in several skill areas, although for the content area with the largest baseline gap (economic evaluation), we saw no improvement. Although deficits in EBDM competencies among state-level practitioners appear to be narrowing over time (25), interventions like ours probably can narrow the gap more rapidly. The skill areas of interest were derived from a systematic process (26) and are essential for making use of many online tools and toolkits for chronic disease control (eg, the *Community Guide*, Cancer Control P.L.A.N.E.T.).

In a systematic review of dissemination studies of cancer prevention in community settings (5), the role of organizational factors in the uptake of evidence-based interventions was scarcely examined. We sought to increase the variety of organization-level variables. Our interventions did not result in significant improvements in measures of organizational capacity. The exception was for the 4-item factor on access to evidence and skilled staff. Organizational change is difficult and requires long-term commitment. It is possible that the interventions in our study were not intensive enough to result in measureable organizational change in some variables. Several studies have shown a high rate of turnover in state public health agencies (27). This ever-changing workforce may make it difficult to develop and maintain an organizational climate and culture supportive of EBDM.

Limitations of this study should be noted. It was difficult to gather objective data on practitioner or agency performance. Although data were well-tested psychometrically, we relied on self-reported (perceived) data on individual-level and organization-level outcomes. We assessed no direct chronic disease outcomes (eg, does greater use of research by practitioners lead to better chronic disease outcomes?), yet a substantial body of literature shows that the variables we measured on EBDM lead to better performance (13). Performance over time was probably improving in our control group given that many programs funded by the Centers for Disease Control and Prevention now require grantees to implement EBDM. Although it is established that individuals influence organizations and the reverse (12), our finding that only 1 of 4 organization-level outcomes was affected by our intervention suggests that more intensive interventions and longer time periods may be needed to change an organization’s climate and culture. Given that our intervention group included only 6 states, our findings may not be generalizable to all states.

This study should be considered first-generation research and can be viewed in the context of the growing literature on dissemination and implementation research (12). Several topics deserve future consideration among practitioners and researchers. First, more tailored, active approaches are warranted. It is unclear whether our study approach was intensive enough to sustain positive changes. In addition, larger effects for subgroups (eg, master’s degree–trained individuals) suggest approaches may need to be adapted for various staff categories. The skill sets for health department staff members may differ from those needed among partners outside of a public health agency. Second, there is a need for better measures of EBDM. One of the greatest needs among chronic disease control practitioners is how to better assess organizational capacity (28). Most existing measures focus on ultimate outcomes, such as change in health status. Most existing measures of capacity have not been tested adequately for reliability and predictive validity (29). Third, capacity building needs to occur in the context of staff turnover. The rate of turnover among participants in our study was substantial, suggesting that frequent exposure to EBDM processes may be needed as new staff members are hired and trained. Fourth, the lack of change in some skill areas (eg, economic evaluation) may call for more intensive skill building or seeking out partners (eg, university staff) to help with more complex content areas. Fifth, more attention is needed on driving organizational change. Changing organizational culture and climate to an environment supportive of EBDM takes time and concerted effort (30). The intervention activities in our study may not have been intensive enough to foster measureable change in organizations, especially considering the heterogeneity in organizations.

To control chronic disease at a population level, EBDM requires a complex set of individual skills and organizational capacity. Our findings suggest several dissemination interventions that should be considered by practitioners as they seek to apply EBDM in their agencies to ultimately benefit the populations they serve.

## Appendix

**Table:** Appendix Table 1. State Health Department Capacity-Building Activities^a^ for Evidence-Based Decision Making (EBDM) in 6 US States, 2014–2016

Domain	Activity	Description
Accreditation	Accreditation preparations	State health assessment and plan, formalized decision making, documentation of evidence, documentation reviews, site visit, approval
Workforce development	EBDM training	In-state, in-person multiday training in EBDM skills, 9 modules, as initial study intervention
	Supplemental brief EBDM skill trainings	Provided by study team or state chronic disease unit, in-person or webinar, as part of this study, with 3 states emphasizing evaluation skills
	Non-study national trainings	Hosted in-person EBDM-related skill trainings by national organizations and/or encouraged out-of-state training beyond those required by funders
	Quality improvement	Quality improvement or performance management trainings, guidance
	New employee EBDM orientation	Via archived webinars or course materials, facilitated discussions, meetings
Leadership, management supports	Chronic disease leadership teams expect EBDM	Leaders and supervisors continually ask “what is the evidence?,” communicate EBDM expectations to staff, champion EBDM, encourage use of data for decision making, encourage skill building
	Use of data for decision making	Use data to prioritize programs, develop work plans, and monitor progress; share performance measures, data on intranet or centralized data systems
	Centralized data systems	Dashboard development to prioritize, measure, and track objectives and link to evidence base; share performance measures and data
	Meetings incorporate EBDM	Work unit and cross-section meetings address EBDM, present evidence, plans (in leadership and in training)
	Performance reviews and EBDM	Work unit employee evaluations include objectives on EBDM learning and application
	Hiring practices address EBDM	Job descriptions, interview questions address EBDM; hire people with public health competencies; hire specialty staff including evaluators and epidemiologists
	Participatory decision making	Staff and partner input obtained, sharing of information for decision making
	Common language for EBDM	Creating and using common EBDM language across program areas
	Administrative reorganization for coordination	Organizational restructuring at the unit or section levels to increase coordination across programs and conduct joint projects across programs
Organizational climate	EBDM engrained	EBDM an embedded inseparable aspect of day-to-day work; strong expectation from leadership; high priority
	Learning orientation	Culture supports professional development and ongoing learning, providing links to webinars, bringing in guest speakers
Relationships and partnerships	Partnerships with in-state universities	Ongoing partnering for evaluation, trainings, internship placement
	Partner technical assistance and training	Telephone and in-person guidance for partners’ evidence-based work plans, evaluation, logic models; provide EBDM trainings to partners
	Relationship building	Active steps to build or maintain positive partner relationships with open communication, trust, mutual respect, ensuring partner engagement and coalition development
Financial practices	Performance-based contracting	Funded partners required to implement evidence-based approaches as prescribed or selected from a menu, with performance objectives, work plans, and evaluation; holding contracted partners accountable for evidence-based interventions
	Proposals approved internally for EBDM before submission to funder	State health department pre-approval process for grant applications to funders with requirements to show objectives, evidence basis, performance measures, evaluation plan

^a^ Not all states participated in all activities.

**Table:** Appendix Table 2. Outcome Measures to Assess Evidence-Based Decision Making Capacity and Supports in 12 States, 2014–2016

Outcomes (Dependent Variables)	Variable Calculation	No. of Items	Item Type	Item (or Sample Item)
**Individual-level capacity**				
**EBDM skill gaps summary**	Sum of 10 calculated gaps	10	Likert 11-point scale	Score for perceived importance of each skill minus score for perceived work unit availability of each skill
**Skill gap**				
Prioritization	Perceived importance minus availability	1	Likert 11-point scale	Prioritization: Understand how to prioritize program and policy options
Adapting interventions				Adapting interventions: Understand how to modify programs and policies for different communities and settings
Quantifying the issue				Quantifying the issue: Understand the uses of descriptive epidemiology (eg, concepts of person, place, time) in quantifying a public health issue
Evaluation designs				Evaluation designs: Understand the different designs that are useful in program or policy evaluation
Quantitative evaluation				Quantitative evaluation: Understand the uses of quantitative evaluation approaches
Qualitative evaluation				Qualitative evaluation: Understand the value of qualitative evaluation approaches (eg, focus groups, key informant interviews)
Economic evaluation				Economic evaluation: Understand how to use economic data in the decision making process
Action planning				Action planning: Understand the importance of developing an action plan for how to achieve goals and objectives
Community assessment				Community assessment: Understand how to define the health issue according to the needs and assets of the population/community of interest
Communicating research to policy				Communicating research to policy makers: Understand the importance of effectively communicating with policy makers about public health issues
**Use of research evidence**	Mean of responses	6	Frequency 4 categories	How often do you use research evidence to: Write a grant application Plan or conduct a needs assessment Select policies, programs, or other interventions Justify selection of interventions to funders, agency leadership, or external partners Evaluate interventions Develop materials for local public health, partners
**Organization-level capacity**				
Access to evidence and skilled staff	Factor created in exploratory factor analysis (EFA)	4	Likert 7-point scale	Agreement with statements: My work unit has access to current research evidence for EBDM Informational resources are available to my work unit to promote the use of EBDM My work unit currently has the resources (eg, staff, facilities, partners) to support application of EBDM The staff in my work unit has the necessary skills to carry out EBDM
Program evaluation	Factor created in EFA	3	Likert 7-point scale	Agreement with statements: My work unit plans for evaluation of interventions before implementation My work unit uses evaluation data to monitor and improve interventions My work unit distributes intervention evaluation findings to other organizations
Supervisory expectations	Factor created in EFA	3	Likert 7-point scale	Agreement with statements: My direct supervisor expects me to use EBDM My direct supervisor recognizes the value of management practices that facilitate EBDM My performance is partially evaluated on how well I use EBDM in my work
Participatory decision making	Factor created in EFA	3	Likert 7-point scale	Agreement with statements: When decisions are made within my work unit, program staff members are asked for input Information is widely shared in my work unit so that everyone who makes decisions has access to all available knowledge My work unit engages a diverse external network of partners that share resources for EBDM
